# Novel germline *CDK4* mutations in patients with head and neck cancer

**DOI:** 10.1186/1897-4287-10-11

**Published:** 2012-08-29

**Authors:** Maimoona Sabir, Ruqia Mehmood Baig, Ishrat Mahjabeen, Mahmood Akhtar Kayani

**Affiliations:** 1 Cancer Genetics Lab, Department of Biosciences, COMSATS Institute of Information Technology, Park Road Chak shahzad, Islamabad, Pakistan

**Keywords:** CDK4, germ line mutations, SSCP, squamous cell carcinoma of head and neck, Pakistani population

## Abstract

**Background:**

Cyclin-dependent kinase 4 (*CDK4*) together with its regulatory subunit cyclin D1, governs cell cycle progression through G_1_ phase. Cyclin-dependent kinase inhibitors, including p16^INK4A^ in turn regulate *CDK4*. In particular, deregulation of the p16/CDK4/cyclin D1 complex has been established in a variety of human tumors including gliomas, sarcomas, melanoma, breast and colorectal cancer. However, changes in *CDK4* have rarely been observed.

**Method:**

In this study we used a combination of PCR-SSCP and direct sequencing for mutational screening of *CDK4*. DNA was isolated from peripheral blood leukocyte of patients with squamous cell carcinoma of head and neck, for screening germline mutations in coding regions of *CDK4*.

**Results:**

Variations observed in exon 2 and 5 were three missense mutations, g5051G > C (Ser52Thr), g5095G > C (Glu67Gln), g5906C > A, g5907C > G (Pro194Ser) and novel frame shift mutations g7321_23delTGA, g7121_7122insG, g7143delG in exon 7 and 3′UTR respectively.

**Conclusion:**

In conclusion, two novel mutations were found in N terminal domain which indicates that *CDK4* mutation may play a major role in the development and progression of squamous cell carcinoma of head and neck.

## Background

Cyclin-dependent kinases (*Cdks*) are serine/threonine kinases that regulate progression through cell cycle. *CDK4* and *CDK6* act early in the cell cycle and are involved in the transition from G_1_ to S phase [1]. Loss of G_1_ control in cell cycle appears to be an important step contributing to tumorigenesis [2]. D-type cyclin and their kinase partners, *CDK4* and *CDK6*, coordinately phosphorylate the Rb protein, thereby releasing the transcription factor at G_1_ and then progressions into the S phase occurs [3]. In addition to role in cell cycle, there is increasing evidence that *Cdks*, as well as cyclin and cyclin-dependent kinase inhibitors are important for other cellular functions, including cytoskeleton rearrangement and cell migration [4]. *CDK4* is a potential oncogene, located on chromosome 12q13; mechanisms of activation could include gene amplification, over-expression, decreased degradation, and activating point mutations. The *CDK4* is amplified and over expressed in a number of human tumors including the gliomas, sarcomas, breast tumors and colorectal carcinomas [5]. In humans, rare point mutations in the *CDK4* have been described worldwide [6]. All *CDK4* reported mutations are located in exon 2, which codes for the p16^INK4A^ binding site [7]. Derailments of Rb pathway caused either by lack of Rb gene (pRb1-CycinD1-Cdk4/6-p16^INK4^) expression and over expression of *Cdks* are implicated in the deregulation of cell cycle machinery, resulting in uncontrolled growth, tumor heterogeneity, invasion and metastasis [8].

Aim of study was to investigate a possible disruption of Rb suppression pathway by germline mutational analysis in the p16^-^binding and cyclin D1 binding domain of *CDK4* in squamous cell carcinoma of head and neck in Pakistani population. Furthermore, to determine association of novel mutations in *CDK4* and risk of squamous cell carcinoma of head and neck, we performed polymerase chain reaction, single strand conformation polymorphism (PCR-SSCP) and sequence analysis.

## Materials and methods

### Sample collection and clinical data

The present study was conducted with a prior approval from ethical committees of both department and hospitals. Blood samples from total of 380 patients with histological confirmed squamous cell carcinoma of head and neck, including oral cavity, pharynx and larynx were collected from National Oncology and Radiotherapy Institute (NORI), Pakistan Institute of Medical Sciences (PIMS) and Military Hospital (MH). A total of 350 age, gender, and ethnicity matched cancer free healthy individuals were selected as controls. Patients and controls suffering from any other familial disease (diabetes, blood pressure and cardiovascular impairment) were excluded from this study. After obtaining informed consent, all individuals were personally interviewed using a specifically designed questionnaire.

### DNA extraction

Blood samples from patients and normal individuals (control), belonging to the same age and gender were collected in tubes containing EDTA and stored at 4°C. DNA was extracted from white blood cells, using standard phenol-chloroform extraction method [9,10] and stored at -20°C for further processing. Electrophoresis was performed on isolated DNA in 1% ethidium-bromide stained agarose gel and photographed (BioDoc Analyze Biometra).

### Polymerase chain reaction (PCR)

Human *CDK4* sequence was taken from Ensemble. Primers were designed by using PRIMER 3 INPUT SOFTWARE version 0.4.0 (Table 1). Coding regions and their exon/intron boundaries of approximately 60 bp sequence of were investigated to identify any splice site variation as well. Each PCR reaction was performed in a 20 μl reaction mixture containing approximately 20 ng of genomic DNA templates, 2 μl (10 mM) of each primer, 0.24 μl (25 mM) of dNTP, 2 μl (10x) PCR buffer and 0.2 μl (5u/μl) of Taq polymerase. PCR profile consisted of an initial melting step of 94°C for 5 min, 35 cycles of 94°C for 45 s, annealing temperature for 1 min and 72°C for 1 min and a final extension step of 72°C for 10 min and hold at 4°C.

**Table 1:** List of primers with annealing temperature and product size (bp)

**Exon No**	**Primer sequence (Forward) F**	**Primer sequence (Reverse) R**	**Product size (bp)**	**Annealing temperature °C**
1	TCACGTGCCCAGAACGTC	CTCATTCCTGGGAAGGGACT	266	56
2	AAGCGACTTTTGGTGATAGGA	TTACTCCCCACGCCCAAC	308	54
3	GAGAGGCCATGTTGGGTTAA	TCCACCTCTCAATGCCTACC	222	58
4	TCTGGGATTTCAGGTATGGTG	ATTGCTACGGGCAATCACTC	295	58
5	GTGTTTCATGGTAACCCATGG	TTTATGAACAAGCGATTTGGG	219	58
6	TCTTTGAAAATGACTGCTACCTT	CCTGGGTTCAGCAGAAAGAG	156	58
7	AGGACCCTCCTGACCAGAGT	CTTTCCCTGTGCCCACAG	205	60
8	TCATGGTTTTCTGACCTTTGC	GCCCTCTCAGTGTCCAGAAG	190	58

### Mutational screening and sequence analysis

Amplification products were resolved on a 2% ethidium bromide containing agarose gel along with 100 bp DNA ladder. PCR products were analyzed by single stranded conformational polymorphism (SSCP) [11] and results were analyzed with the gel documentation system (BioDocAnalyze, Biometra). Samples with an altered mobility patterns were reamplified and than analyzed by direct sequencing to confirm and characterize the nature of mutations. Sequence analysis was carried out by MCLab (USA). Control (normal) samples were also sequenced along with cases to avoid false negative results and check the quality of sequencing.

### Statistics and bioinformatic analysis

*χ*2-test with Fisher exact test was used to evaluate the differences in selected demographic variables by using the SPSS 17.0 software, odd ratios (OR) and 95% confidence interval (CI) were calculated. Bioinformatic analysis for homology modeling was performed using ClustalW 2.1 multiple sequence alignment and UCSF Chimera 1.5.3 program.

## Results

### Patient characteristics

The features of our population are listed in Table 2. These 380 patients have mean age of 38.1 years at diagnosis. The mean age of controls (35 ±12.23 years) was slightly different from cases (38 ± 16.35), but the difference was not significant (*p* < 0.86). Statistically significant increase in incidence of head and neck cancer was observed in age group of <40 years as compare to age group > 40 (*p* < 0.05) (Table 2).

**Table 2:** Characteristics of head and neck cancer patients

**Variables**		**Cases N = 380 (%)**
Gender		
	Male	196 (51.5%)
	Female	184 (48.5%)
Age at diagnosis		
	Mean (±S.D)	38 (±16.35)*
	<40	271 (71%)
	>40	109 (29%)
Family history of cancer		
	Yes	65 (17%)
	No	315 (83%)
Cancer type*		
	Oral cavity	224(59%)
	Pharynx	97(25.5%)
	Larynx	59(15.5%)
Smoking status*		
	Non smokers	238 (62.5%)
	Smokers	142 (37.5%)

* Mean age, 38 years (±S.D).

* Cancer type include squamous cell carcinoma of oral cavity, pharynx and larynx.

### PCR-SSCP and direct sequence analysis

**Figure 1:**
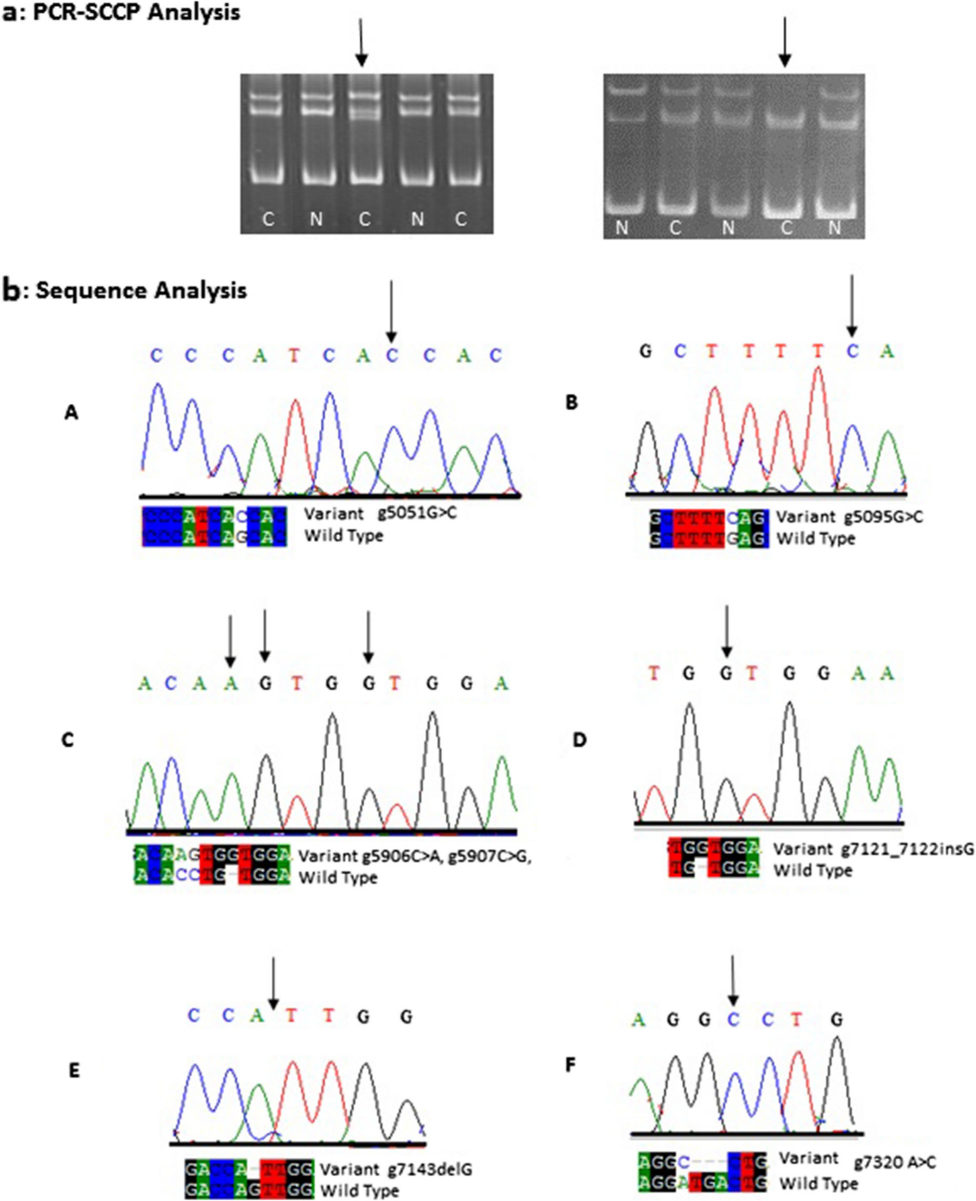
**a: Mutations in*CDK4*detected by PCR-SSCP analysis in head and neck cancer samples, digital image of an ethidium bromide stained 8% non denaturing polyacrylamide gel showing band pattern.** Arrow head indicates change in band shift. C represents HNC cases and N represents normal samples. **b**: Sequencing electropherogram of mutations in CDK4: (**A**) Missense g5051G > C mutation, (CM994495) in exon 2 showing G to C substitution resulting in change of DNA sequences from AGC to ACC encoding amino acid threonine instead of serine, (**B**) Missense mutation, g5095G > C (Novel) in exon 2 showing G to C substitution resulting in change of DNA sequence from GAG to CAG encoding the amino acid glutamine instead of glutamate, (**C**) Missense mutations, g5906C > A, g5907C > G in exon 5 changing the DNA sequence from CCT to AGT encoding the amino acid serine instead of proline, (**D**) Frame shift mutation in 3′UTR as a result of insertion of nucleotide G, (g7121_7122insG), (**E**) Frame shift mutation in 3′UTR as a result of deletion of nucleotide G (g7143delG), (**F**) Missense mutation g7320A > C and frameshift mutation g7321_23delTGA in exon 7.

To investigate whether alterations of the *CDK4* were present in the squamous cell carcinoma of head and neck, we screened the coding regions (exons 1–8) as well as intron/exon boundaries of *CDK4* using PCR-SSCP. Figure 1 (a & b) summarizes the results of PCR-SSCP and direct sequence analysis. In this study different types of mutations were identified in exon 2, 5, 7 and 3′UTR. No alteration in exon 1, 3, 4, 6 and 8 of *CDK4* was detected in any of cases.

Study revealed a reported missense mutation g5051G > C (CM994495) in exon 2 show G to C substitution resulting in change of nucleotide sequences from AGC to ACC, encoding amino acid threonine instead of serine. A novel missense mutation g5095G > C resulting in change of nucleotide sequence from GAG to CAG, encoding the amino acid glutamine instead of glutamate. Study includes other novel missense mutations, g5906C > A and g5907C > G, in exon 5 changing the nucleotide sequence from CCT to AGT, encoding the amino acid serine instead of proline. A novel missense g7320A > C mutation and a frame shift mutation g7321_23delTGA was also detected in exon 7. Two novel frame shift mutations were observed in 3′UTR as a result of insertion of nucleotide G (g7121_7122insG) and deletion of nucleotide G (g7143delG) (Table 3). No such mutation was present in control samples. Oral cavity cases have more mutation compare to pharyngeal and laryngeal carcinoma but difference was statistically non significant [*p* = 0.13, OR 84.0, 95% CI (65.69 to 233.69)]. Significant difference was observed in smokers and non-smokers patients having missense and frame shift mutations [*p* = 0.03, OR 1.84, 95% CI (1.33 -2.54)] (Table 4). To verify whether the Arg24Cys mutation in the *CDK4* was present in squamous cell carcinoma, we analyzed a portion of exon 2 of the *CDK4*, where this mutation mapped by PCR-SSCP and subsequent sequence analysis. Both SSCP and sequence analysis showed that Arg24Cys mutation was not present in any of our cases.

**Table 3:** **Frequency of** ***CDK4*** **mutations in squamous cell carcinoma of oral cavity, pharynx & larynx**

**Region in gene**	**Mutations**	**Mutation type**	**Change in amino acid**	**Frequency of mutations**
Exon 2	g5051G > C*	Missense*	Ser52Thr	0.08
Exon 2	g5095G > C	Missense	Glu67Gln	0.13
Intron 3	g5427delA	-	-	0.14
Exon 5	g5906C > A, g5907C > G	Missense	Pro194Ser	0.19
Exon 7	g7320 A > C, g7321_23delTGA	Frame shift	-	0.15
3′ UTR	g7121_7122insG	Frame shift	-	0.16
3′ UTR	g7143delG	Frame shift	-	0.09

* Reported mutation with CM994495 coding unknown *http://www.ensembl.org/Homo_sapiens/Variation/Summary?db=core;g=ENSG00000135446;r=12:58142034–58149796;v=CM994495;vdb=variation;vf=44643002.

**Table 4:** **Distribution of** ***CDK4*** **mutations with area of cancer and smoking status**

**Region in gene**	**Mutations/change in amino acid**	**Area of cancer**			**Smoking status**	
		**Oral cavity (N = 224)**	**Pharynx (N = 97)**	**Larynx (N = 59)**	**Non smoker (N = 238)**	**Smokers (N = 142)**
Exon 2	g5051G > C	24	19	06	19	30
Exon 2	g5095G > C	22	15	08	22	23
Intron 3	g5427delA	18	11	02	17	14
Exon 5	g5906C > A, g5907C > G	15	08	04	15	12
Exon 7	g7320 A > C, g7321_23delTGA	20	03	01	13	11
3′ UTR	g7121_7122insG	27	12	07	22	24
3′ UTR	g7143delG	23	05	02	12	18
Total		149	73	30	120	132
		*p =* 0.13			*p =* 0.03	
		OR 84.0, 95% CI (65.69 to 233.69)			OR 1.84, 95% CI (1.33 -2.54)	

N = number of cases.

Odd ratio (OR).

95% confidence interval (CI).

## Discussion

**Figure 2:**
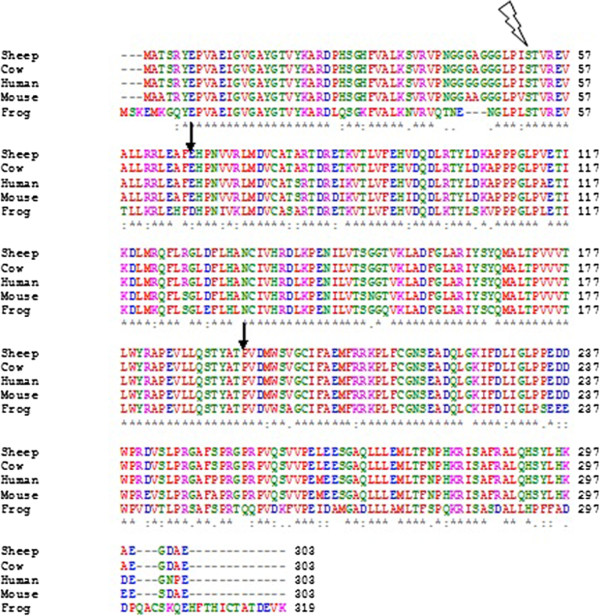
**Multiple sequence alignment of*CDK4*protein generated with ClustalW 2.1.** The locations of two of the point mutations reported here are marked with arrows, and one previously reported mutation, Sequences from five different species were included in the alignment: Human [P11802], mouse [P30285], sheep [B2MVY4] cow bovine [Q32KY4] and frog[Q91727].

The Rb pathway has been shown to be frequently altered in human cancers [12-15]. *CDK4* is a member of the Ser-Thr protein kinase family and its catalytic domain extends from amino acid 6 to 295. *CDK4* consists of eight exons, of which the start codon is located in the beginning of exon 2 and the stop codon in the beginning of exon 8 [16]. Multiple sequence alignment of *CDK4* protein was generated with ClustalX, including sequences from five different species was constructed. As shown in Figure 2, the human and sheep cdk4-protein sequences have a higher level of identity than the average human-mouse sequence identity (97% versus 94%).

**Figure 3:**
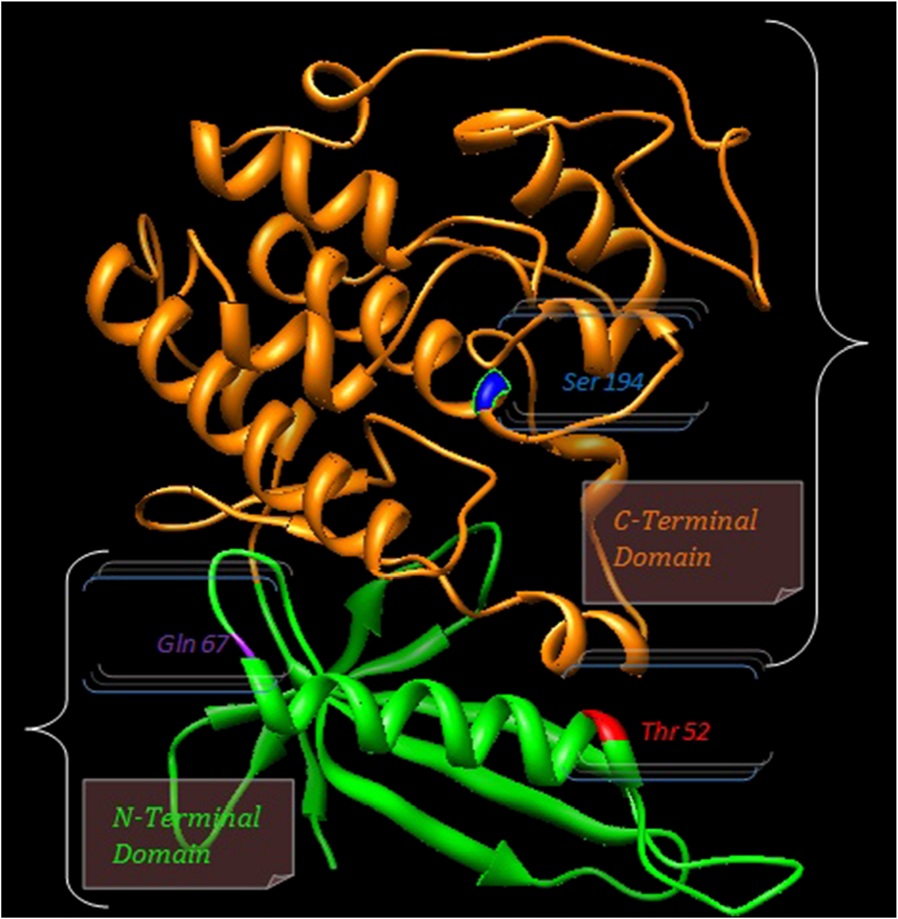
***CDK4*protein structure indicating the N-terminal and C-terminal domain.** The mutations Ser52Thr, Glu67Gln and Pro194Ser are represented in the structure, using The UCSF Chimera 1.5.3 program.

In present study germline mutational analysis of *CDK4* revealed two missense mutations, Ser52Thr and Glu67Gln on exon 2. However, to date, only two mutations in this region, an arginine-to-cysteine missense mutation [17] and an arginine-to-histidine mutation in the germ line of one French melanoma-prone family [6] in codon 24 and 22 have been reported respectively. This mutation at codon 24, as a result of change of a single nucleotide (CGT to TGT) change arginine to cysteine (R24C) is an activating mutation, as it results in growth advantages, because it prevents p16 binding [18]. A prior study found that mutations that decreased cyclin binding of *CDK4* by greater than 80% also decreased kinase activity [19]. Generally, the level of cyclin binding of the various *CDK4* mutants correlates well with kinase activity, and the sequences involved in p16^INK4a^ binding are a subset of those involved in cyclin D1 binding [20]. Figure 3 shows *In silico* structural model analysis of *CDK4* protein using the UCSF Chimera 1.5.3 program revealed that Ser52Thr and Glu67Gln mutations are located in N terminal domain contain αC helix (aa 1–96). That compromise of PISTVRE sequence is important for protein function and binding of cyclin D [21].

In our study three novel frame shift mutations were found on exon 7 and 3′UTR. Frame shift mutation observed in coding region g7321_23delTGA is changing the whole downstream sequence of gene. As a result translation of wrong reading frame continues and the resultant premature RNA stability is compromised so either mRNA will not stable or protein degradation happen [22]. Although genetic alterations in 3*′* UTR sequences can modify the binding properties of *trans*-acting factors and lead to deregulation in protein production [18]. But most studies have sought to identify mutations in the coding region and very few naturally occurring mutations in non coding areas have been described to date. Pakistan harbors vast genetic, ethnic, cultural, social and life style diversity. In present study a group is characterized by onset of cancer frequently before the age of 40 year. Clinically, one key feature of genetic bases of cancer is early age at onset. Similar trends have been reported for familial melanoma, breast and colon cancers. Early disease onset has been associated with an increased prevalence of germline mutations [23].

Amplification and over expression of *CDK4* has been detected in sarcoma and glioma, but in carcinoma the picture seems to be unclear. Only sporadic data are available in carcinoma where very low percentage of amplification was reported [24]. *CDK4* is altered in melanoma patients by a miscoding mutation (Arg24Cys) that blocks binding of INK4 inhibitors. However, the causal role of these alterations in tumor development is difficult to assess [25]. Present study revealed that there was no germline mutation in codon 24 and 22 of *CDK4* and these results support the findings of previous studies [20,26,27]. The potentially dominant Arg24Cys mutation of *CDK4* was not detected in Indian patients with squamous cell carcinoma of head and neck [8]. *CDK4* mutations reported in the literature are rare and little is known about the functional implications of these changes [28]. Study using *in vitro* site-directed mutagenesis analyzed that disruption of either codon 22 or codon 24 effectively abrogates interaction with cyclin Dl and p16^INK4a^[19]. *In vivo* analysis of patients with the germ-line Arg24Cys mutation, revealed no documented changes in p16/CDKN2A binding site of *CDK4*[29].

## Conclusion

In conclusion Arg24Cys mutation plays a key role in different cancers but in the current study we were unable to detect this mutation. This suggests that *CDK4* novel germline mutations observed in study may play a different and important role in squamous cell carcinoma of head and neck cancer in Pakistani population. Potential of these novel mutations in head and neck carcinogenesis need to be explored in order to ascertain there potential importance.

## Competing interests

The authors declare that they have no competing interests.

## Authors’ contributions

MS: Study concept and design; acquisition of data; drafting of the manuscript; genetic and bioinformatics analysis; statistical analysis. RMB: Contribution to drafting of the manuscript; interpretation of data. IM: Acquisition of data; collection of samples and interpretation of data. MAK: Study supervision. All authors read and approved the final manuscript.
